# Erythema marginatum may be urticaria

**DOI:** 10.1016/j.jdin.2024.04.001

**Published:** 2024-04-18

**Authors:** Daphne Thampy, Domenica Del Pozo, Jordan T. Hyde, Sylvia Hsu

**Affiliations:** Department of Dermatology, Temple University Lewis Katz School of Medicine, Philadelphia, Pennsylvania

**Keywords:** bacterial infections, erythema marginatum, infectious disease, medical dermatology, rheumatic fever, urticaria

*To the Editor:* Erythema marginatum is a figurate erythema classically associated with rheumatic fever, a delayed sequela of *Streptococcus pyogenes* infection. However, comparison of clinical presentation and histopathology of urticaria suggests these diagnoses are the same condition. Urticaria is often included in the differential diagnosis for erythema marginatum, but the criteria necessary for establishing the distinction between them remain unclear.

Erythema marginatum was first described in 1831 by Bright and was later found to be a skin manifestation of rheumatic fever in 1881 by Barlow and Cheadle.^1^ It is classified as one of the major criteria used in the diagnosis of rheumatic fever, although observed in <6% of cases.^2^ Meanwhile, urticaria affects 15% to 25% of the population at some point in their lifetime.^3^ Urticaria is often associated with a recent bacterial or viral infection. Streptococcal infection is reported as the cause of 17% of urticaria cases in children, the population most affected by rheumatic fever.^4^ The paucity of cases of erythema marginatum reported with rheumatic fever is most likely due to the indistinguishable presentations between erythema marginatum and urticaria, a common occurrence secondary to streptococcal infection.

Erythema marginatum and urticaria are characterized by a transient cutaneous eruption, presenting as a migratory, annular, and polycyclic erythematous eruption.^5^ Although some describe the defining feature between erythema marginatum and urticaria to be the presence of a nonpruritic rash, this is not universal, as cases of erythema marginatum with pruritus have been described and cases of urticaria without pruritus are not uncommon. Furthermore, histopathology of both conditions demonstrates a sparse superficial perivascular lymphocytic infiltrate with interstitial neutrophils and eosinophils suggesting a shared immunologic pathway. The diagnosis of erythema marginatum and urticaria is made clinically. There are no known clinical or histopathologic characteristics specific to either one that allow for differentiation.

However, this is not to say the presence of a cutaneous eruption associated with a recent streptococcal infection is a benign finding. In some cases of rheumatic fever, a rash can mark the onset of carditis. Similar to urticaria, erythema marginatum has also been associated with angioedema, in which erythema marginatum is identified as a frequent prodrome. Regardless, the skin finding is usually self-limited, and management remains the same—address the underlying condition. Although there are no differentiating factors between these diagnoses, identification of urticaria may provide a diagnostic clue in the progression of an untreated disease course.

Given the widespread overlap between erythema marginatum and urticaria, it is likely that these conditions are the same entity. The shared clinical and histopathologic features challenge the traditional boundaries of these diagnoses, which is mostly dependent on the presence or lack of pruritus but is seen in both conditions. The diagnosis of erythema marginatum is rarely discussed as a diagnostic consideration today due to the rarity of rheumatic fever but is merely accepted as a distinct cutaneous eruption still present in most modern textbooks. We suggest that the cutaneous eruption associated with rheumatic fever be classified and managed as urticaria. However, further investigation is needed to elucidate the differences between these 2 diagnoses.

## Conflicts of interest

None disclosed.
